# Developing methods to detect and diagnose chronic traumatic encephalopathy during life: rationale, design, and methodology for the DIAGNOSE CTE Research Project

**DOI:** 10.1186/s13195-021-00872-x

**Published:** 2021-08-12

**Authors:** Michael L. Alosco, Megan L. Mariani, Charles H. Adler, Laura J. Balcer, Charles Bernick, Rhoda Au, Sarah J. Banks, William B. Barr, Sylvain Bouix, Robert C. Cantu, Michael J. Coleman, David W. Dodick, Lindsay A. Farrer, Yonas E. Geda, Douglas I. Katz, Inga K. Koerte, Neil W. Kowall, Alexander P. Lin, Daniel S. Marcus, Kenneth L. Marek, Michael D. McClean, Ann C. McKee, Jesse Mez, Joseph N. Palmisano, Elaine R. Peskind, Yorghos Tripodis, Robert W. Turner, Jennifer V. Wethe, Jeffrey L. Cummings, Eric M. Reiman, Martha E. Shenton, Robert A. Stern, for the DIAGNOSE CTE Research Project Investigators, Charles H. Adler, Michael L. Alosco, Rhoda Au, Laura Balcer, Sarah Banks, William Barr, Charles Bernick, Sylvain Bouix, Robert C. Cantu, Kewei Chen, Michael J. Coleman, Jeffrey L. Cummings, David W. Dodick, Lindsay Farrer, Jennifer Fitzsimmons, Yonas Geda, Judith Goldberg, Robert Helm, Keith A. Johnson, Douglas I. Katz, Ivan Kirov, Inga K. Koerte, Neil Kowall, Alexander P. Lin, Yvonne Lui, Daniel S. Marcus, Kenneth L. Marek, Megan Mariani, Charles Marmar, Michael McClean, Ann C. McKee, Jesse Mez, Jason Miller, Joseph N. Palmisano, Ofer Pasternak, Elaine R. Peskind, Hillary Protas, Eric Reiman, Aaron Ritter, Martha E. Shenton, Robert A. Stern, Yi Su, Yorghos Tripodis, Robert W. Turner, Jason Weller, Jennifer V. Wethe

**Affiliations:** 1 Boston University Alzheimer’s Disease Research Center, Boston University CTE Center, Department of Neurology, Boston University School of Medicine, Boston, MA USA; 2 Boston University CTE Center, Boston University School of Medicine, Boston, MA USA; 3 Department of Neurology, Mayo Clinic College of Medicine, Mayo Clinic Arizona, Scottsdale, AZ USA; 4 Departments of Neurology, Population Health and Ophthalmology, NYU Grossman School of Medicine, New York, NY USA; 5 Cleveland Clinic Lou Ruvo Center for Brain Health, Las Vegas, NV USA; 6 Boston University Alzheimer’s Disease Research Center, Boston University CTE Center, Framingham Heart Study, and Slone Epidemiology Center, Boston, MA USA; 7 Departments of Neuroscience and Psychiatry, University of California, San Diego, CA USA; 8 Department of Neurology, NYU Grossman School of Medicine, New York, NY USA; 9 Psychiatry Neuroimaging Laboratory, Department of Psychiatry, Brigham and Women’s Hospital, Harvard Medical School, Boston, MA USA; 10 Boston University Alzheimer’s Disease Research Center, Departments of Neurology and Neurosurgery, Boston University School of Medicine, Boston, MA USA; 11 Psychiatry Neuroimaging Laboratory, Brigham and Women’s Hospital, Boston, MA USA; 12 Departments of Medicine (Biomedical Genetics), Neurology, Ophthalmology, Epidemiology, and Biostatistics, BU Schools of Medicine and Public Health, Boston, MA USA; 13 Alzheimer’s Disease and Memory Disorders Program, Department of Neurology, Barrow Neurological Institute, Phoenix, AZ USA; 14 Department of Neurology, Boston University School of Medicine, Boston, MA USA; 15 Center for Clinical Spectroscopy, Department of Radiology, Psychiatry Neuroimaging Laboratory, Department of Psychiatry, Brigham and Women’s Hospital, Harvard Medical School, Boston, MA USA; 16 Department of Radiology, Washington University School of Medicine, St. Louis, MO USA; 17 Institute for Neurodegenerative Disorders, Invicro, LLC, New Haven, CT USA; 18 Department of Environmental Health, Boston University School of Public Health, Boston, MA USA; 19 Boston University Alzheimer’s Disease Research Center, Boston University CTE Center, Framingham Heart Study, Department of Neurology, Boston University School of Medicine, Boston, MA USA; 20 Biostatistics and Epidemiology Data Analytics Center (BEDAC), Boston University School of Public Health, Boston, MA USA; 21 VA Northwest Mental Illness Research, Education, and Clinical Center, VA Puget Sound Health Care System, Department of Psychiatry and Behavioral Sciences, University of Washington School of Medicine, Seattle, WA USA; 22 Department of Biostatistics, Boston University School of Public Health, Boston, MA USA; 23 Department of Clinical Research & Leadership, The George Washington University School of Medicine & Health Sciences, Washington, DC USA; 24 Department of Psychiatry and Psychology, Mayo Clinic School of Medicine, Mayo Clinic Arizona, Scottsdale, AZ USA; 25 Chambers-Grundy Center for Transformative Neuroscience, Department of Brain Health, School of Integrated Health Sciences, University of Nevada Las Vegas, Las Vegas, NV USA; 26 Banner Alzheimer’s Institute, University of Arizona, Arizona State University, Translational Genomics Research Institute, and Arizona Alzheimer’s Consortium, Phoenix, AZ USA; 27 Psychiatry Neuroimaging Laboratory, Departments of Psychiatry and Radiology, Brigham and Women’s Hospital, Harvard Medical School, Boston, MA USA; 28 Boston University Alzheimer’s Disease Research Center, Boston University CTE Center, Departments of Neurology, Neurosurgery, and Anatomy & Neurobiology, Boston University School of Medicine, Boston, MA USA; 29 Department of Neurology, University of Washington, Seattle, WA USA; 30 Departments of Anatomy & Neurobiology and Neurology, Boston University School of Medicine, Boston, MA USA; 31 Department of Epidemiology, Boston University School of Public Health, Boston, MA USA; 32 Encompass Health Braintree Rehabilitation Hospital, Braintree, MA USA; 33 cBRAIN, Department of Child and Adolescent Psychiatry, Psychosomatics, and Psychotherapy, Ludwigs-Maximilians-Universität, Munich, Germany; 34 VA Boston Healthcare System, Boston, MA USA

**Keywords:** Neurodegenerative disease, Biomarkers, Chronic traumatic encephalopathy, Cognitive function, Concussion, Football, Head trauma, MRI, MRS, National Football League, College football, Neuroimaging, Repetitive head impacts, Traumatic brain injury, Subconcussion, Traumatic encephalopathy syndrome, Positron emission tomography, Tau, Remote assessment

## Abstract

**Background:**

Chronic traumatic encephalopathy (CTE) is a neurodegenerative disease that has been neuropathologically diagnosed in brain donors exposed to repetitive head impacts, including boxers and American football, soccer, ice hockey, and rugby players. CTE cannot yet be diagnosed during life. In December 2015, the National Institute of Neurological Disorders and Stroke awarded a seven-year grant (U01NS093334) to fund the “Diagnostics, Imaging, and Genetics Network for the Objective Study and Evaluation of Chronic Traumatic Encephalopathy (DIAGNOSE CTE) Research Project.” The objectives of this multicenter project are to: develop in vivo fluid and neuroimaging biomarkers for CTE; characterize its clinical presentation; refine and validate clinical research diagnostic criteria (i.e., traumatic encephalopathy syndrome [TES]); examine repetitive head impact exposure, genetic, and other risk factors; and provide shared resources of anonymized data and biological samples to the research community. In this paper, we provide a detailed overview of the rationale, design, and methods for the DIAGNOSE CTE Research Project.

**Methods:**

The targeted sample and sample size was 240 male participants, ages 45–74, including 120 former professional football players, 60 former collegiate football players, and 60 asymptomatic participants without a history of head trauma or participation in organized contact sports. Participants were evaluated at one of four U.S. sites and underwent the following baseline procedures: neurological and neuropsychological examinations; tau and amyloid positron emission tomography; magnetic resonance imaging and spectroscopy; lumbar puncture; blood and saliva collection; and standardized self-report measures of neuropsychiatric, cognitive, and daily functioning. Study partners completed similar informant-report measures. Follow-up evaluations were intended to be in-person and at 3 years post-baseline. Multidisciplinary diagnostic consensus conferences are held, and the reliability and validity of TES diagnostic criteria are examined.

**Results:**

Participant enrollment and all baseline evaluations were completed in February 2020. Three-year follow-up evaluations began in October 2019*.* However, in-person evaluation ceased with the COVID-19 pandemic, and resumed as remote, 4-year follow-up evaluations (including telephone-, online-, and videoconference-based cognitive, neuropsychiatric, and neurologic examinations, as well as in-home blood draw) in February 2021.

**Conclusions:**

Findings from the DIAGNOSE CTE Research Project should facilitate detection and diagnosis of CTE during life, and thereby accelerate research on risk factors, mechanisms, epidemiology, treatment, and prevention of CTE.

**Trial registration:**

NCT02798185

**Supplementary Information:**

The online version contains supplementary material available at 10.1186/s13195-021-00872-x.

## Background

Chronic traumatic encephalopathy (CTE) is a neurodegenerative disease that has been neuropathologically diagnosed in former contact sport athletes, military combat veterans, and other individuals exposed to repetitive head impacts [1–9]. Although CTE is often portrayed as a new disease, its history dates back to the 1920s and 1930s, with descriptions of “punch drunk” boxers [10] and “dementia pugilistica” [11], with the term “chronic traumatic encephalopathy” first used in publications in the 1940s [12, 13]. CTE began to receive significant lay and scientific attention in 2005, following a report of neuropathological evidence of hyperphosphorylated tau (p-tau) in an irregular pattern in a deceased former National Football League (NFL) player who had ante-mortem cognitive and neuropsychiatric symptoms [5]. CTE has since been neuropathologically diagnosed in hundreds of deceased American football players [1–3], in addition to other contact and collision sport athletes (e.g., ice hockey, soccer, rugby) [2, 8, 14] and military veterans [4]. Given the millions of active and former contact sport athletes, military service members, and veterans, CTE is potentially a major public health concern. However, disease incidence and prevalence remain unknown due to the inability to detect and diagnose CTE during life.

### Neuropathology of CTE

In 2013, McKee et al. examined the immunocytochemical characteristics of 68 cases (including 50 American football players) with autopsy-confirmed CTE and proposed neuropathological diagnostic criteria for CTE in addition to a 4-stage classification scheme to grade the pathological severity of p-tau [2, 15]. As part of the National Institute of Neurological Disorders and Stroke (NINDS)-funded “Understanding Neurologic Injury in Traumatic Encephalopathy (UNITE)” study [16], two consensus meetings have been convened to define the neuropathological diagnostic criteria for CTE [17, 18]. The pathognomonic lesion is now defined as p-tau aggregates as neurofibrillary tangles (NFT) in neurons, with or without p-tau in astrocytes, deposited around small blood vessels, in an irregular pattern at the depths of the cortical sulci, with the focus in superficial cortical layers. The panels concluded that the pattern of p-tau in CTE is distinct from that of any other neurodegenerative disease [17, 18]. The molecular structure of tau filaments in CTE has also since been shown to be unique [19] [20] [21]. Although CTE is a mixed three and four microtubule-binding domain repeat (3R and 4R) tauopathy, similar to AD, recent research has shown that the CTE tau isoforms shift across disease severity, from 4R to 3R, with 4R isoforms found primarily in astrocytes [22]. Unlike in AD, amyloid-beta protein (Aβ) deposits in CTE are not an early feature, they are not diagnostic, and they tend to accumulate with advancing age as a co-morbidity. When present in CTE, the Aβ plaques tend to be diffuse and not neuritic [23]. CTE is nonetheless frequently co-morbid with other neurodegenerative (e.g., Lewy body disease) and non-neurodegenerative conditions (e.g., white matter rarefaction, arteriolosclerosis) [3, 24–26].

### Risk factors for CTE

The primary risk factor for CTE is exposure to repetitive head impacts and the resulting repeated concussions and subconcussive trauma (i.e., head impacts that result in neuronal injury but do not cause immediate symptoms) [3, 9, 24, 27–30]. Repetitive subconcussive head impacts play a prominent role in the development of this disease [31, 32].The *duration* of American football play has been identified as a strong predictor of the risk and severity of CTE neuropathology [30]. Small autopsy case series provide suggestive links between soccer and rugby play and CTE [8, 14]. These associations between repetitive head impacts and neuropathology have been complemented by in vivo research studies that link exposure to repetitive head impacts with later-life cognitive and neuropsychiatric symptoms [24, 27, 33, 34]. However, not all individuals who are exposed to repetitive head impacts will develop CTE or other long-term neurological disorders. Risk modifiers may include severity and nature of repetitive head impact exposure (e.g., frequency [27, 35], type of contact sport played [36–38], younger age of first exposure [39–44]), lower cognitive reserve [45], cerebrovascular health [24, 46], and/or genetic make-up [47–49].

### Clinical presentation of CTE and traumatic encephalopathy syndrome

CTE is a neuropathological diagnosis and cannot currently be diagnosed during life because its clinical presentation has been—until recently—ill-defined, and because validated in vivo biomarkers for the detection of CTE neuropathology do not yet exist [50]. To date, the clinical presentation of CTE has been described primarily through the use of retrospective telephone interviews with next-of-kin and other informants of brains donors with autopsy-confirmed CTE. These studies have reported a constellation of progressively worsening and non-specific cognitive impairments (particularly in executive functions and episodic memory), poor regulation or control of emotions and/or behavior (including impulsivity, explosiveness, rage, and/or emotional lability), and, in some instances, parkinsonism and motor neuron disease [3, 47, 51]. Provisional clinical research diagnostic criteria for CTE have been proposed [52, 53], including the traumatic encephalopathy syndrome (TES) research criteria published in 2014 [54]. Since the time of the original 2014 publication, the research diagnostic criteria for TES have been used in several studies. Their validity in predicting neuropathological CTE diagnoses has recently been reported along with an item-level analysis suggesting that cognitive symptoms—more than neuropsychiatric features—are particularly valuable in predicting CTE pathology [55]. These findings informed new NINDS Consensus Diagnostic Criteria for TES that have recently been published [56].

Consistent with the clinical diagnosis of other neurodegenerative diseases [57–60], an accurate diagnosis of CTE during life will require validated biomarkers of the underlying pathophysiology of the disease. There have been several preliminary studies that have examined both *specific* (e.g., those that measure regional p-tau pathology) and *non-specific* (e.g., general neurodegeneration) biomarkers of CTE. Examples of potential supportive or non-specific fluid biomarkers of CTE include cerebrospinal fluid (CSF) and plasma markers of neurodegeneration (e.g., total tau [t-tau] [35, 61, 62], neurofilament light [NfL] chain protein [63]) and microglial activation (e.g., soluble triggering receptor expressed on myeloid cells 2 [sTREM2]) [61], as well as measures of tau in exosomes isolated from plasma and proteomic profiling of plasma and CSF extracellular vesicles [64–66]. Candidate neuroimaging biomarkers for the detection of *non-specific* neurodegenerative changes of CTE have recently been reviewed [67, 68] and include the following: cavum septum pellucidum (a common gross neuropathological finding in CTE [1, 2]) on structural T1-weighted magnetic resonance imaging (MRI) [69–72]; frontotemporal and medial temporal lobe atrophy on structural T1-weighted MRI [73–79]; decreased cortical thickness on T1-weighted MRI [44]; white matter alterations on diffusion tensor imaging (DTI) [42, 74, 80–84]; cerebral hypoperfusion and functional hypoactivity on single photon emission computerized tomography [85]; arterial spin labeling [86] and functional MRI (fMRI) [87]; and neurochemical alterations on magnetic resonance spectroscopy (MRS) [88–90].

Efforts are underway to identify *specific* biomarkers of CTE. Amyloid positron emission tomography (PET) imaging is a neuropathologically validated biomarker for the early detection, tracking, and diagnosis of neuritic Aβ deposition, one of the cardinal neuropathological features of AD [58, 91]. Although postmortem studies of CTE have found diffuse Aβ plaques, evidence of neuritic plaques has primarily been found in late-stage disease [3, 15, 23]. It would be unlikely for amyloid PET tracers to demonstrate significant binding in CTE, as they bind preferentially to neuritic plaques [91]. More recently, PET imaging of paired helical filament tau has become a neuropathologically validated biomarker for the detection, tracking, and diagnosis of AD-related tau tangle deposition. For instance, the PET radiotracer flortaucipir (AV1451; T807) demonstrates high affinity binding to the mixed 3R/4R tau isoforms in AD [92]. Flortaucipir’s binding to other tauopathies with primarily 4R isoforms has not been as promising [93, 94]. Although CTE and AD have similar (3R/4R) tau isoforms, there are differences at the molecular level [19], and, as mentioned earlier, in CTE the ratio of 3R:4R differs across disease stage and between neuronal and glial tau [22].

Stern et al. studied flortaucipir PET in 26 symptomatic former NFL players (ages 40–69) and 31 same-age asymptomatic men without a history of traumatic brain injury (TBI) [95]. At a group level, the former players had significantly higher flortaucipir uptake in superior frontal, medial temporal, and parietal regions, compared to the asymptomatic men without TBI; however, the uptake levels were not as high as seen in individuals with AD. Although the level of flortaucipir uptake in the three brain regions was significantly associated with the number of years playing football, it was not significantly associated with cognitive and neuropsychiatric test scores. Importantly, the former players did not have elevated florbetapir levels compared to the unexposed group, and only one former NFL player had slightly elevated florbetapir standardized uptake value ratio (SUVR), indicating that the former NFL players’ cognitive impairment was not due to AD. Based on these findings and other in vivo and neuropathological studies [78, 96, 97], the utility and specificity of flortaucipir for the detection of CTE paired helical filament tau is unclear.

### The DIAGNOSE CTE Research Project

In December 2015, NINDS funded a 7-year, multicenter grant proposal, “Chronic Traumatic Encephalopathy: Detection, Diagnosis, Course, and Risk Factors,” submitted by our multidisciplinary team of investigators, under the leadership of four co-principal investigators (co-PIs; RAS [contact PI], JLC, EMR, MES). This is now referred to as the “Diagnostics, Imaging, and Genetics Network for the Objective Study and Evaluation of Chronic Traumatic Encephalopathy (DIAGNOSE CTE) Research Project.” The specific aims of the DIAGNOSE CTE Research Project are listed in Table 1. The primary endpoints of the study are to characterize the clinical presentation of CTE and to identify in vivo biomarkers that can support a “probable CTE” diagnosis. There were several guiding principles that were followed throughout the development of the grant proposal and study design, including (1) CTE is viewed as a *neurodegenerative* disease; (2) although the necessary risk factor for the development of this tauopathy appears to be a history of repetitive head impact exposure, CTE itself should not be confused with TBI or considered the aggregate effect of multiple symptomatic concussions; (3) to conduct a study of the clinical presentation, diagnostic criteria, biomarkers, and risk factors of CTE requires expertise across many disciplines, including Neurology, Neuropsychology, Psychiatry, Neuroimaging, Molecular Medicine, Neuropathology, Exposure Science, Genetics, Biostatistics, Epidemiology, Computer Science, and Sociology; and (4) a team science approach is required, breaking down typical academic and institutional “silos” to conduct the best research in the most efficient manner.

**Table 1:** Specific aims of the DIAGNOSE CTE Research Project

**Aim 1**: To collect and analyze neuroimaging and fluid biomarkers for the in vivo detection of CTE.	
**Aim 2**: To characterize the clinical presentation of CTE.	
**Aim 3**: To examine the progression of CTE in a longitudinal study.	
**Aim 4**: To refine diagnostic criteria for the clinical diagnosis of CTE.	
**Aim 5**: To investigate genetic and head impact exposure risk factors for CTE.	
**Aim 6**: To share project data with researchers across the country and abroad.	

The objective of this paper is to describe the rationale, methodology, and design of the DIAGNOSE CTE Research Project. We provide updates on the current status of the project and conclude with methodological considerations, and discussion of the expected impact of the project results, as well as the infrastructure and resources created to support further studies.

## Methods

### Infrastructure overview

The DIAGNOSE CTE Research Project is a 7-year (now expected to be 8-year following COVID-19 pandemic-related modifications described below) study that involves more than 40 investigators from 12 research institutions across the USA (Supplementary Table 1, Additional File 1). There is a 7-member External Advisory Board (Supplementary Table 2, Additional File 1) that meets annually with the co-PIs and the NINDS Scientific Program Official. The project is overseen by an Executive Committee consisting of the co-PIs, Participant Evaluation Site PIs, NINDS Scientific Program Official, Advisory Board Chair (*ex officio* member), and Team Leaders for the seven multidisciplinary/multi-institutional study teams: *Data; Neuroimaging; Fluid Biomarkers; Clinical Outcomes; Diagnostic Criteria; Risk Assessment; and Diversity, Equity, and Inclusion (DEI).* Investigator meetings are held annually.

### Sample selection

The targeted sample and sample size was 240 men, ages 45–74, divided into three groups based on the extent of exposure to repetitive head impacts: 120 former professional football players (PRO); 60 former college football players (COL); and a group of 60 participants with no history of participation in organized contact/collision sports, combat military service, or known concussion or other TBI, herein referred to as the unexposed comparison group (UE). Inclusion and exclusion criteria are summarized in Table 2. There were no enrollment criteria for presence or severity of cognitive or neuropsychiatric symptoms or of degree of functional independence for PRO and COL participants (other than the requirement to have adequate decisional capacity to provide consent for research at baseline). All participants were required to have a study partner who knew them well and with whom they had frequent communication. Based on telephone screening, UE participants were required to be asymptomatic.

**Table 2:** Inclusion and exclusion criteria

	Inclusion criteria	Exclusion criteria
All participants (N = 240)	Male; age 45–74; no contraindications for MRI, LP, or PET procedures; English as primary language; agree to all procedures; willingness to have and availability of a study partner (i.e., informant who knows the participant well, speaks or visits with participant at least weekly for a minimum of 6 months, is > 18 years of age, has English as primary language) who agrees to participate in a telephone interview and respond to a series of online questionnaires.	History of clinical stroke or significant neurologic condition; vision or hearing impairment severe enough to compromise neuropsychological testing; impaired decisional capacity to provide informed consent to participate in study; currently clinically significant infectious disease, endocrine or metabolic disease, pulmonary, kidney or liver impairment, or cancer; body weight > 400 pounds
Unexposed group (UE) (N = 60)	No history of participation in organized contact/collision sports^a^ or military combat service or training; asymptomatic, based on telephone screening questions^b^ (asked of both participant and informant) regarding current mood, behavior, or cognitive symptoms, as well as functional dependence; have at least 2 years of post-secondary education at a 4-year accredited college/university, or have an associate’s degree	History of TBI or concussion; history of formal diagnosis or treatment of psychiatric illness or cognitive impairment; body mass index (BMI) < 24
Former college football player group (COL) (N = 60)	Played > 6 years of organized American football, with > 3 years at the college varsity level; played one of the following positions^c^ while in college: offensive lineman, defensive lineman, linebacker, offensive back or receiver, or defensive back (individuals whose primary position was kicker or quarterback were not included); no organized football or other contact/collision sport following college	
Former professional football player group (PRO) (N = 120)	Played ≥ 12 years of organized football, including > 3 in college and > 3 seasons in the NFL; played one of the following positions^c^ while in the NFL: offensive lineman, defensive lineman, linebacker, offensive back or receiver, or defensive back (individuals whose primary position was kicker or quarterback were not included)	

^a^The unexposed participants must have never participated in any of the following organized sports at any level: American football, ice hockey, rugby, soccer (under age 14 allowed only if no heading the ball), lacrosse, wrestling, boxing, gymnastics, martial arts, and/or kickboxing

^b^Telephone screening includes modified versions (for telephone) of the AD8 Dementia Screening Interview [98] and the Cognitive Change Index [99] (Items 1–12), as well as additional screening questions regarding mood, behavior, and cognitive functioning. These were administered to potential participants as well as their study partners

^c^Following enrollment, when there was a question about an individuals’ position data reported during in-person interview, data from online resources were used to confirm position(s). Some positions may vary in future exposure modeling studies based on specific percentages of downs played at each position reported

### Sample size determination and power

The sample sizes were determined to assure statistical power of 80% or greater, to detect moderate effect sizes, and assuming a significance level of 0.05 with two-tailed significance tests, for key hypotheses for Specific Aims 1–5. Power calculations were performed and based on effect sizes from our previous research. The difference in CSF p-tau/tau ratio between PRO and UE groups was used as an example of the biomarkers evaluated in Aim 1. Preliminary unpublished results from the NINDS-funded DETECT study (PI: RAS) show a difference of 0.15 (SD of 0.04) in CSF p-tau/tau ratio between former NFL players and UE participants. Our sample size has a power of > 99% to detect differences between the symptomatic PRO and COL participants (n = 120) compared to asymptomatic PRO and COL participants and UE participants (n = 120) in p-tau/tau ratio. To illustrate the power for Aim 3, the longitudinal differences between symptomatic PRO participants compared to asymptomatic PRO and UE participants for the Trail Making B test were used. Gavett et al. showed differences ranging from 35 to 60 s over a 3-year follow-up between people with normal cognition and participants with mild cognitive impairment or dementia [100]. Our sample will have 94% power to detect similar differences in a 3-year period among symptomatic PRO participants compared to asymptomatic PRO and UE participants, assuming 30% attrition.

### Recruitment and retention

A Recruitment and Retention Coordinator and research assistant oversaw an extensive national recruitment campaign (Supplementary Material, Additional File 1). Recruitment efforts were aimed at enrolling across a continuum of symptom severity, from asymptomatic to mildly symptomatic to dementia, rather than to any specific level of impairment. Interested potential participants underwent a telephone screening interview by Coordinating Center staff, using a script approved by the Institutional Review Board (IRB) at BU Medical Campus (BUMC). Structured and semi-structured questions were asked about current mood, behavior, and cognitive symptoms, as well as functional status. An additional telephone screening was conducted with an informant/study partner, using similar questions and assessments. The Recruitment and Retention Coordinator determined the Participant Evaluation Site most appropriate for the participant (based on balancing the number of participants across sites, travel distance, and available schedules). To maximize sample retention over the follow-up period, participants are telephoned annually by project staff and sent birthday and holiday cards. A study-wide newsletter is published quarterly and distributed to all participants electronically. For participants with dementia, an additional annual call is made to the participant or (with permission) to a study partner/informant to assess the participant’s status and improve retention.

### Study procedures

A centralized project Coordinating Center is located at Boston University (BU) School of Medicine (BUSM). There are four Participant Evaluation Sites: (1) *Boston* (BUSM, with MRI scans conducted at Brigham and Women’s Hospital [BWH]); (2) *Las Vegas* (Cleveland Clinic [CC] Lou Ruvo Center for Brain Health); (3) *New York* (New York University [NYU] Langone Health); and (4) *Scottsdale/Phoenix* (Mayo Clinic Arizona, with PET scans conducted at Banner Alzheimer’s Institute [BAI] in Phoenix). All participants received a baseline evaluation at one of the four Participant Evaluation Sites. Baseline evaluations included neurocognitive testing, assessment of functional status, neuropsychiatric questionnaires, neurological assessment (including standardized motor examination, headache severity and sleep-related symptoms measurement, and an olfaction test), MRI (including structural, diffusion, functional, and neurochemical), two PET scans (with florbetapir amyloid and flortaucipir tau tracers), lumbar puncture (LP; for CSF banking and biomarkers), blood draws (for banking, biomarkers, and DNA extraction), and saliva samples (for banking and biomarkers). In the original design, the PRO and UE groups would return for a 3-year, in-person follow-up evaluation. COL participants would not be evaluated at follow-up because their inclusion was for head impact exposure risk modeling at baseline (Aim 5) and to assure a large baseline sample size with adequate variability of clinical presentation (Aims 1 & 2).

It was required that all participants have adequate decisional capacity at the time of their baseline visit to participate. Because some participants have mild dementia, specific procedures were conducted to assure appropriate decisional capacity to consent to research participation. Some participants who reported functional difficulties (n = 16) were accompanied by their study partner or other care partner. All participants’ travel expenses (and that of a care partner if required) were paid by the study and each participant received $500 compensation for completion of the 3-day evaluation. Participants were informed during screening, at the time of consenting, and subsequent to their study visit, that they could receive a summary of non-experimental study results (including standardized neuropsychological testing and neuropsychiatric questionnaires, neurological examination, clinical reads of the structural MRI, clinical laboratory blood tests, electrocardiogram) and/or have the results sent to their primary healthcare provider. If requested, they could discuss results of the non-experimental assessments with one of the co-PIs (RAS). At all times, participants were informed orally and in written reports that the results should not be used for clinical or medico-legal decision-making. At the time of study initiation, and throughout the baseline evaluation period, flortaucipir (tau) PET imaging was in human trials and viewed as one of the experimental assessments. It received FDA approval 3 months following completion of baseline examinations for the evaluation of AD, with specific package insert wording that it is not indicated for the evaluation of CTE. Florbetapir (amyloid) PET imaging received FDA approval prior to study initiation for patients being evaluated for AD and is considered non-experimental for specific clinical purposes. For this study, the co-PIs, with input from external experts, chose not to disclose florbetapir results, though this does not necessarily represent the standard for the field moving forward.

### Data collection

#### Clinical evaluations

Each baseline study visit was conducted over a 3-day period and each follow-up visit was planned to take place over a two- or three-day period. Tables 3 and 4 list the clinical exams and measures that were administered. Comprehensive semi-structured interviews for all participants were performed and supplemented by online questionnaires in order to collect data on demographics (e.g., age, education, racial and ethnic identity); psychosocial and lifestyle history (e.g., exercise, occupational and educational attainment, early childhood zip code or equivalent, parents’ educational attainment); medical, neurological, and psychiatric history (including substance use and performance enhancing drug use); family history of psychiatric and neurological conditions; athletic history (e.g., age of first exposure, level(s) and duration of play, position(s) played, era of play); military history; and concussion and TBI history. For the COL and PRO participants, involvement in current or pending litigation involving neurologic consequences of playing American football was also queried. Participants had vital signs (e.g., blood pressure, pulse, height and weight measurement) assessed by a registered nurse. Safety procedures (e.g., blood draw for platelet count and other clotting tests, and ECG for abnormal heart rhythms and/or clinically significant cardiovascular disease) were reviewed by a qualified clinician to ensure participants were eligible for the LP and flortaucipir PET scan, respectively. During the study visit, study partners were emailed a survey link to a web-based Research Electronic Data Capture (REDCap) system to complete standardized measures and a self-report questionnaire on the presence and onset of cognitive, behavior, and/or mood problems, as well as an assessment of functional status. If the informant accompanied the participant to the visits, she/he was asked to complete the online questionnaires prior to returning home. All informants were also interviewed by telephone to provide additional history and to clarify history provided by the participant.

**Table 3:** Baseline and 4-year remote follow-up neurologic, neurocognitive, functional, and health assessments

Domain	Test/Instrument	Baseline	Follow-up
Neurologic exam	Semi-structured clinical examination of cranial nerves, sensory functions, muscle strength, fasciculations, and reflexes	I	–
Motor	International Parkinson and Movement Disorder Society-Unified Parkinson’s Disease Rating Scale (MDS-UPDRS) [101]	I	V
	Timed Up and Go (TUG) [102]	I	–
Effort (symptom validity)	Test of Memory Malingering (TOMM) [103] [104]	I	V
Estimated premorbid intelligence	Wide Range Achievement Test-Fourth Edition (WRAT-4) Word Reading []	I	–
Cognitive screening	Montreal Cognitive Assessment (MoCA) [106, 107]	I	T,V
Attention, visual scanning, and psychomotor speed	Symbol Digit Modalities Test [108]	I	V
	Trail Making Test Part A [109]	I	–
	Unified Data Set (UDS) Number Span Test [110]	I	T
Executive function	Behavior Rating Inventory of Executive Function-Adult Version (BRIEF-A) Global Executive Composite (GEC; Participant and Informant) [111]	R	R
	BRIEF-A Metacognition Index (MI; Participant and Informant) [111]	R	R
	Phonemic Fluency - Controlled Oral Word Association Test (COWAT) [112]	I	T
	Golden Stroop Color and Word Interference [98]	I	–
	Neuropsychological Assessment Battery (NAB) Mazes [99, 113]	I	–
	Trail Making Test Part B [109]	I	–
	NAB Categories [99]	–	V
	Cambridge Neuropsychological Test Automated Battery (CANTAB) One Touch Stockings of Cambridge (OTS) [114, 115]	–	C
	CANTAB Spatial Working Memory (SWW) [114, 115]	–	C
	CANTAB Cambridge Gambling Task (CGT) [114, 115]	–	C
Learning and memory	Brief Visuospatial Memory Test – Revised (BVMT-R) [104]	I	V
	UDS Craft Story [110]	I	T
	NAB List Learning [99]	I	T
	CANTAB Paired Associate Learning (PAL) [114, 115]	–	C
	CANTAB Pattern Recognition Memory (PRM) [114, 115]	–	C
Language	Category (Semantic) Fluency - Animals [112, 116]	I	T
	Category (Semantic) Fluency - Vegetables [112, 116]	–	T
	UDS Multilingual Naming Test (MINT) [110]	I	V
Visuospatial ability	Judgment of Line Orientation (JOLO) [117]	I	V
	BVMT-R Copy [104]	I	V
Emotional facial perception	CANTAB Emotional Recognition Task (ERT) [114, 115]	–	C
Dementia severity	Functional Activities Questionnaire (FAQ; Participant and Informant) [118]	R	R
	Quick Dementia Rating System (QDRS; Participant and Informant) [119]	R	R
Sleep	Mayo Sleep Questionnaire (MSQ; Participant and Informant) [120]	R	R
	Epworth Sleepiness Scale [121]	R	R
Olfactory	Brief Smell Identification Test (B-SIT) [122]	I	–
Pain	Headache Impact Test (HIT-6) [123]	R	R
	Brief Pain Inventory (BPI) [124, 125]	P	P
Substance use	Alcohol Use Disorders Identification Test: Interview Version (AUDIT) [126]	I	T
	Modified National Institute on Drug Abuse (NIDA) Alcohol, Smoking and Substance Involvement Screening Test (ASSIST) version 2.0 [127]	I	T
Health status	EQ-5D-5L Health Questionnaire [128]	P	P
	National Health and Nutrition Examination Survey (NHANES) Physical Activity and Physical Fitness Questionnaire (PAQ) [129]	I	T
	NHANES Weight History Questionnaire [130]	I	T

Note: *I* administered in-person; *V* Administered over HIPAA compliant Zoom Video conferencing (modified from original in-person method); *T* administered over telephone; *R* administered over REDCap online data capture forms; *P* administered on paper form; *C* administered online using CANTAB Web-Based Testing platform

**Table 4:** Baseline and 4-year remote follow-up neuropsychiatric instruments

Domain	Instrument
Affective lability	Center for Neurologic Study – Lability Scale (CNS-LS) [131]
Behavior	Barratt Impulsivity Scale-11 (BIS-11) [132]
	BRIEF-A Behavioral Regulation Index (BRI; Participant and Informant) [111]
	Brown-Goodwin Lifetime History of Aggression (BGLHA) [133]
	Buss-Durkee Hostility Inventory (BDHI) [134]
	Mild Behavioral Impairment – Checklist (MBI-C; Participant and Informant) [135]*
	Neuropsychiatric Inventory – Questionnaire (NPI-Q; Informant) [136]
	State-Trait Anger Expression Inventory-II (STAXI-II) [137]
Depression, anxiety, apathy, other	Apathy Evaluation Scale (AES) [138]
	Beck Anxiety Inventory (BAI) [139]
	Beck Depression Inventory-II (BDI-II) [140]
	Beck Hopelessness Scale (BHS) [141]
	PTSD Checklist – Civilian Version (PCL-C) [142]
	Sheehan-Suicidality Tracking Scale (S-STS) [143]
	University of California, Los Angeles Loneliness Scale (UCLA) [144]

*Added for follow-up evaluations only

Clinical measures (see Tables 3 and 4) were selected, in part, to assure harmonization with data sharing platforms, such as the Federal Interagency Traumatic Brain Injury Research (FITBIR) system and the National Alzheimer Coordinating Center (NACC). Many instruments and methodologies that overlap with the NINDS Common Data Elements (CDE) and/or the NACC Uniform Data Set (UDS) v.3.0 (the latter used by all of the National Institute on Aging-funded AD Research Centers) [110] were selected. Measures include those that assess clinical domains relevant to the features described in neuropathologically confirmed cases of CTE [2, 3, 47, 54] and were part of the 2014 TES research diagnostic criteria [54].

To assure standardization of the administration and scoring of clinical evaluations across sites and examiners, extensive training procedures were employed. Neurologists administering the Movement Disorder Society (MDS)-Unified Parkinson’s Disease Rating Scale (UPDRS) [101] completed formal online training offered through MDS. A comprehensive neuropsychological test administration and scoring manual was developed and deployed to all sites, along with an accompanying training video of a full test administration, including several demonstrations of how to respond to and score incorrect or unusual responses. All staff administering the neurocognitive tests were certified (and re-certified annually) in test administration and scoring via mock training videos that were reviewed and certified by two licensed clinical neuropsychologists at the Coordinating Center (MLA, RAS).

#### Neuroimaging

Neuroimaging protocols include structural T1- and T2-weighted MRI, diffusion MRI (dMRI), resting-state fMRI, MRS, and molecular imaging with two PET tracers, florbetapir and flortaucipir. During the pre-enrollment period, BWH neuroimaging investigators and Invicro (a research-dedicated organization that collaborates on large-scale diagnosis, progression, and disease monitoring trials, providing molecular imaging services, including florbetapir and flortaucipir) created study-specific image acquisition sequences and technical operations manuals and developed and implemented training and setup procedures for the MR and PET centers, respectively, at each of the Participant Evaluation Sites. Details on the neuroimaging processing and analysis are provided in the Supplementary Material (Additional File 1).

##### MRI

MRIs across all four sites were conducted on Wide Bore 3 T scanners (Siemens Skyra, Erlangan, Germany; software version VE11) using a 20 channel head coil in order to accommodate the wide range of participant sizes. The goal of the MRI sequence selection was to obtain the most advanced images consistent with other large multi-site studies (e.g., Alzheimer’s Disease Neuroimaging Initiative), and which could be acquired at each site within a reasonable time period to limit participant burden. The acquisition included sequences for anatomical images, as well as diffusion MRI (dMRI) and resting-state fMRI. High-resolution (1 × 1 × 1 mm^3^) 3D T1-weighted images using MPRAGE with an inversion time of 1100 ms were acquired, as were high-resolution (1 × 1 × 1 mm^3^) 3D T2-weighted images and fluid attenuated inversion recovery (FLAIR) sequences. The dMRI has a multi-shell design with 73 acquisitions spread over 5 shells (4 b = 0, 3 b = 200, 6 b = 500, 30 b = 1000, and 30 b = 2500 s/mm^2^). Images have a 2 × 2 × 2 mm^3^ resolution and 73 slices. The resting-state fMRI acquisition was an echo-planar imaging (EPI) acquisition with 3.5 × 3.5 × 3.5 mm^3^ resolution, with 37 slices, TR of 2.5 s, repeated 149 times.

##### MRS

2D-chemical shift imaging (CSI) was acquired using the localized semi-adiabatic spin-echo refocusing (semi-LASER) with Gradient-Offset independent Adiabaticity Wurst modulation (GOIA-W) pulses and spiral encoding (853-ms duration, 12-kHz bandwidth, 90° flip angle, 160 mm field of view, 1.5-s repetition time, and 40-ms echo time) [145]. Interleaved constant-density spirals simultaneously encode one frequency and two spatial dimensions (16 × 16; 3 averages) for a resolution of 1 × 1 × 1.5 cm^3^ and a scan time of 6 min. The 160 × 160 × 15 mm slab was placed across the corpus callosum parallel to the A/P plane. Single voxel spectroscopy (SVS) was acquired using point-resolved spectroscopy (PRESS; TE = 30 ms, TR = 2 s, 2 × 2 × 2 cm^3^, 128 averages; 16 average water reference) in the posterior cingulate gyrus for a scan time of 5 min [146].

##### PET

Participants underwent two PET imaging studies (florbetapir and flortaucipir) at baseline. Tracer doses were requested through Avid Radiopharmaceuticals (Philadelphia, PA, USA) who then ordered the doses from one of several contract manufacturing organizations (usually the most proximate to a site) and coordinated dose shipping and delivery to the four PET centers. The florbetapir protocol was as follows: immediately after a 370 MBq (10 mCi) bolus injection, the participant underwent brain scans consisting of 10 frames, each 1 min in length. Fifty minutes after injection, the participant completed a second 15-min brain scan consisting of three frames, each of which required 5 min.

The use of flortaucipir in this study was carried out through an Investigator Investigational New Drug (IND #131391) from the U.S. Food and Drug Administration. The flortaucipir protocol was as follows: 80 min after a 370 MBq (10 mCi) bolus injection, the participant completed a continuous dynamic 20-min brain scan (four frames, 5 min each). Procedures described by Stern et al. [95] will serve as a guide for initial analyses of florbetapir and flortaucipir images. However, additional analyses of PET amyloid and tau scans will be conducted, consistent with the most current methods and approaches [147–149].

#### Fluid biomarkers

The collection, tracking, banking, and distribution of all fluid biospecimens is done under the direction of the project’s Fluid Biomarker Team leader (ERP) at VA Puget Sound. CSF, blood, and saliva collection and storage complies with the National Institute on Aging Biospecimen Task Force Guidelines and with NINDS Repository Biomarkers Discovery Samples Resource. Education and training were provided at each of the Participant Evaluation Sites (through in-person training, provision of a video DVD [150], and manuals) for the safe, acceptable, and uniform methods for CSF, blood, and saliva collection. The Fluid Biomarker Team provided all sites with prefabricated CSF, blood, and saliva sample collection kits. Sample collection and sample processing procedures are detailed in the Supplementary Material (Additional File 1). An aliquot of whole blood was kept at room temperature and shipped to BUSM the day of collection for DNA extraction for genetic and genomic analyses. All other saliva, blood, and CSF samples were processed, aliquoted, and stored at − 80 °C at the four Participant Evaluation Sites, and then batch shipped on dry ice overnight to VA Puget Sound, where they are stored in two − 70 °C freezers. Banked CSF, blood products, and saliva will be made available to qualified outside investigators.

#### Head impact exposure assessment and modeling

A challenge of evaluating the long-term consequences of repetitive head impacts is that the outcomes are chronic, but the exposures are acute and, in this setting, remote. Each impact is of short duration, can be ambiguous, and rarely quantified. Task-based exposure assessment methods, such as job-exposure matrices, are often utilized to develop retrospective exposure metrics for investigating exposure-disease relationships [151]. The same will be applied to retrospectively estimate repetitive head impact exposure in the COL and PRO groups. A position-exposure matrix (PEM) will be developed. Different football positions (e.g., running back, offensive lineman) experience different impacts in terms of frequency, intensity, location, and type (linear or rotational) [152–154]. These measurements have been collected for over 1.8 million head impacts during games and practices using the Head Impact Telemetry (HIT) System™ [155–159]. Information from this extensive database will be utilized to construct the PEM. The PEM will use the most current HIT data to summarize the variation of impacts by position and level of play. One limitation of this approach is that the NFL has not publicly released, nor have there been published reports of HIT System or other head impact sensor data from NFL players, thus resulting in the need to rely on college player HIT System data in these PEMs. We will combine the PEM with each participant’s football history (i.e., age of first exposure, level[s] and duration of play, position[s] played) to develop participant-specific estimates of cumulative exposure to head impacts [27, 61, 160, 161]. Additional methods of estimating repetitive head impact exposure will be included as they become available. Practical proxies of exposure to repetitive head impacts will be examined, such as years of American football play and age of first exposure to American football, among others. Data on the participant’s self-reported number of concussions [162, 163] and number of episodes of loss of consciousness using the Ohio State University TBI Identification Method-Interview Form [164] were collected as metrics of additional history. The participant-specific exposure estimates will be used to evaluate clinical and biomarker outcomes.

#### Genetics

Whole blood collected at the time of the blood draw was shipped directly to the Molecular Genetics Core at BUSM where DNA was isolated, frozen, and used for apolipoprotein E (APOE) genotyping. We will conduct genome-wide genotyping using the Illumina Global Screening Array (Illumina, Inc., San Diego, CA, USA).

### Multidisciplinary Diagnostic Consensus Conferences (MDCC)

Each month there are two MDCCs held through videoconference and attended by a panel of 16 clinician-investigators, including 8 neurologists, 5 neuropsychologists, 2 psychiatrists, and 1 neurosurgeon, from 7 institutions. Each MDCC is required to have a quorum of one panelist from at least three of the four Participant Evaluation Sites, a minimum of two neurologists and two neuropsychologists, representation from at least two sites outside of BU, and a minimum of five panelists in attendance. At each MDCC, the history and findings from approximately 5–9 participants are presented. Following presentation of the history, course, and test score summaries (including measures of subjective cognitive complaints, functional independence, and sleep, as well as neurocognitive, neuropsychiatric, neurologic, and motor functioning), each MDCC member provides their independent diagnosis of TES, in addition to other clinical disorders due to neurodegenerative diseases using established diagnostic criteria (e.g., mild cognitive impairment [MCI] and AD dementia using the National Institute on Aging – Alzheimer’s Association criteria [165]) and psychiatric disorders based on the Diagnostic and Statistical Manual of Mental Disorders, Fifth Edition (DSM-5) [166]. The MDCC members share and discuss their ratings and adjudicate a final consensus diagnosis (based on majority).

### Modified remote follow-up evaluations

As a result of the COVID-19 pandemic, the project co-PIs, in collaboration with NINDS Program Officials, and with input from the Executive Committee and External Advisory Board, decided that all follow-up evaluations would be changed to entirely remote assessments to maintain the safety of our participants and study staff, while also preserving the scientific integrity of the overall study. Remote assessments were required given that participants are flown from their homes to one of the 4 Participant Evaluation Sites, and the pandemic placed severe restrictions on travel. All follow-up procedures were approved by the BUMC IRB. Each participant is assessed for decisional capacity to provide consent for research participation, using a modification of the University of California, San Diego Brief Assessment of Capacity to Consent (UBACC) [167]. Informed consent forms are signed digitally and, in the case of participants who are determined to lack decisional capacity, their research proxy or legally authorized representative digitally signs the consent form. Each participant receives $325 compensation for completion of all follow-up procedures. The modified remote follow-up evaluation is conducted using three separate platforms: (1) telephone, (2) online, and (3) video. Based on prior Baseline Evaluation experience, all participants have access to a telephone and most have access to an internet-connected desktop or laptop computer. Results from a survey conducted of study participants in the spring of 2020 indicated that a large majority have access to a desktop or laptop computer with a webcam for videoconferencing. Most of the tests included in the remote follow-up evaluation have been found to result in comparable performance when administered in-person or remotely via telephone, online, or videoconference platforms [107, 115, 168–171].

All follow-up participants are interviewed over the telephone to update any history and lifestyle information and to conduct standardized interview-based assessments. In addition, all participants are administered a telephone-based neurocognitive evaluation which includes the telephone modification of the NACC UDS 3.0 cognitive assessment battery (T-Cog, including the Neuropsychological Assessment Battery (NAB) List Learning Test) [99, 110] and the telephone version of the Montreal Cognitive Assessment (MoCA) (T-MoCA) [107]. Participants with access to an internet-connected desktop or laptop computer also complete a battery of web-based computerized cognitive tests from the Cambridge Neuropsychological Test Automated Battery (CANTAB) [114]. Those participants who have a webcam (with proficiency in using these devices determined during a screening prior to follow-up) are administered additional video-based (using the Zoom videoconference platform) neurocognitive tests and also undergo a neurological evaluation, including a modified MDS-UPDRS examination [172, 173], by a board-certified and MDS-UPDRS-trained neurologist who specializes in movement disorders (CHA). Selection of the final battery of neurocognitive measures was made by the full team of project neuropsychologist-investigators to assure that all domains of interest were assessed. All participants and study partners are asked to complete an online REDCap survey to assess cognitive, mood, and behavior difficulties, as well as functional independence, using identical methods employed during baseline evaluations. Follow-up tests and questionnaires, including the modality of assessment, are listed in Tables 3 and 4. The Adverse Childhood Experiences (ACEs) questionnaire [174] was added to the follow-up evaluation to assess childhood factors that may contribute to adult physical and psychological health outcomes.

All consenting participants have a fasting blood draw at their home at the time of their follow-up evaluations. Blood collection and sample preparation is conducted by phlebotomists from ExamOne (a Quest Diagnostics Company, Lenexa, KS) who undergo study-specific training and who are provided with prefabricated blood collection and sample preparation supplies, along with a manual and infographic detailing all procedures, from the BU Coordinating Center. Whole blood, serum, and plasma samples are prepared and aliquoted, put on dry ice within 90 min of centrifugation, and shipped to VA Puget Sound, where they are banked for biomarker assays and distribution to qualified investigators (see Supplementary Material, Additional File 1, for details).

All participants will receive a follow-up diagnosis 4 years after their initial assessment using the NINDS Consensus Diagnostic Criteria for TES [56] through the same MDCC process as baseline diagnoses.

### Management and sharing of data and biospecimens

The Biostatistics and Epidemiology Data Analytics Center (BEDAC) at the BU School of Public Health provides data management, database and web development, and data analytics for the project (the latter in collaboration with the project’s lead biostatistician and Data Team Leader (YT)). Data are collected using web-based data capture for assessments using REDCap, as well as customized forms for complex data. Common data elements and study-specific data elements are uploaded to FITBIR on a regular basis to allow for data sharing in the latter part of the project. Once baseline data collection was completed, the Data Team developed a web-based data sharing platform, initially for use by project investigators, with the plan for subsequent availability to all qualified researchers (i.e., in the latter part of the project). Based on the specific needs of an investigator, a customized dataset is created using an automated system. Raw imaging data and fluid biosamples will be made available to qualified investigators (see Supplementary Material, Additional File 1).

## Results

### Participants

All Participant Evaluation Site institutions (i.e., BU, CC, Mayo, NYU) and associated sites (BWH, BAI) received approval by their governing IRB by January 2017. All participants provided written informed consent during their baseline visit. Enrollment began in September 2016 and the last baseline evaluation was completed in February 2020. The final analytic sample includes 240 men, ages 45–74, including 120 PRO, 60 COL, and 60 UE participants. Table 5 summarizes sample demographics. An additional 24 participants who underwent some or all baseline evaluations are excluded from all data analyses and subsequent evaluation for a variety of reasons, including UE participants who reported a history of concussion (n = 6) or who were found to have extensive psychiatric history (n = 3) during in-person interview, participants with incomplete biomarker data (n = 10), participants who self-withdrew (n = 2), or were withdrawn by a PI for other reasons (n = 3). Three-year follow-up in-person evaluations (for PRO and UE participants) began in October 2019, with 11 completed by March 6, 2020. Due to the COVID-19 pandemic, all in-person study activities were ceased on March 16, 2020. Follow-up evaluations have shifted to fully remote and are being conducted on all participants, including the COL participants (see below for details).

**Table 5:** Demographic summary of DIAGNOSE CTE Research Project sample at baseline

	Former NFL players (PRO)	Former college football players (COL)	Unexposed (UE)	p value^**a**^
Total N	120	60	60	–
Age, mean (SD) years	59.1 (7.8)	53.5 (7.7)	59.3(8.3)	< 0.01^b^
Age by decade, n (%) years				
45–54	42 (35.0)	44 (73.3)	23 (38.3)	< 0.01
55–64	46 (38.3)	9 (15.0)	15 (25.0)	
65–75	32 (26.7)	7 (11.7)	22 (36.7)	
Body mass index, mean (SD) kg/m^2^	32.0 (4.5)	33.8 (4.8)	30.8 (4.5)	< 0.01^c^
Years of education, mean (SD)	16.6 (1.1)	17.1 (1.0)	17.3 (3.4)	0.38
Level of education, n (%)				
Some college, no degree	15 (12.5)	5 (8.3)	7 (11.7)	< 0.01
Associate degree	2 (1.7)	1 (1.7)	8 (13.3)	
Bachelor’s degree	82 (68.3)	35 (58.3)	23 (38.3)	
Master’s degree	16 (13.3)	17(28.3)	12 (20.0)	
Doctoral degree	5 (4.2)	2 (3.4)	10 (16.7)	
Racial Identity, n (%)				
American Indian or Alaska Native	1 (0.8)	0 (0.0)	0 (0.0)	0.05
Black or African American	51 (42.5)	10 (16.7)	24 (40.0)	
Native Hawaiian or other Pacific Islander	0 (0.0)	0 (0.0)	1 (1.7)	
White	66 (55.0)	48 (80.0)	35 (58.3)	
Multiracial	1 (0.8)	1 (1.7)	0 (0.0)	
Not reported	1 (0.8)	1 (1.7)	0 (0.0)	
Ethnicity, n (%)				
Hispanic or Latino	3 (2.5)	0 (0.0)	0 (0.0)	0.28
Not Hispanic or Latino	117 (97.5)	60 (100.0)	59 (98.3)	
Unknown/not reported	0 (0.0)	0 (0.0)	1 (1.7)	
Marital status, n (%)				
Never married	5 (4.2)	3 (5.0)	15 (25.0)	< 0.01
Married or domestic partnership	88 (73.3)	43 (70.0)	31 (51.6)	
Divorced or separated	23 (19.2)	14 (23.3)	13 (21.7)	
Widowed	1 (0.8)	1 (1.7)	0 (0.0)	
Other	3 (2.5)	0 (0.0)	1 (1.7)	
Employment status, n (%)				
Working full-time	57 (47.5)	40 (66.7)	27 (45.0)	< .01
Working part-time	10 (8.3)	0 (0.0)	11 (18.3)	
Unemployed	1 (0.8)	7 (11.7)	1 (1.7)	
Retired	38 (31.7)	9 (15.0)	21 (35.0)	
Disabled	13 (10.8)	2 (3.3)	0 (0.0)	
Other/refused	1 (0.8)	2 (3.3)	0 (0.0)	
Level of father’s education, n (%)				
Less than high school	24 (20.0)	3 (5.0)	8 (13.3)	0.01
High school diploma or graduate equivalent degree (GED)	43 (35.8)	15 (25.0)	18 (30.0)	
Some college or associate degree	10 (8.3)	11 (18.3)	6 (10.0)	
Bachelor’s degree	15 (12.5)	19 (31.7)	12 (20.0)	
Master’s degree	9 (7.5)	6 (10.0)	10 (16.7)	
Doctoral degree	7 (5.8)	4 (6.7)	3 (5.0)	
Unknown	12 (10.0)	2 (3.3)	3 (5.0)	
Level of mother’s education, n (%)				
Less than high school	19 (15.8)	4 (6.7)	5 (8.3)	0.04
High school diploma or GED	52 (43.3)	21 (35.0)	22 (36.7)	
Some college or associate degree	16 (13.3)	11 (18.3)	11 (18.3)	
Bachelor’s degree	18 (15.0)	17 (28.3)	10 (16.7)	
Master’s degree	6 (5.0)	6 (10.0)	8 (13.3)	
Doctoral degree	1 (0.8)	1 (1.7)	3 (5.0)	
Unknown	8 (6.7)	0 (0.0)	1 (1.7)	
History of learning disability or ADHD, n (%)	9 (7.5)	10 (16.7)	1 (1.7)	0.02
Site evaluated, n (%)				
Boston	26 (21.7)	18 (30.0)	20 (33.3)	0.58
Las Vegas	33 (27.5)	13 (21.7)	11 (18.3)	
New York	28 (23.3)	15 (25.0)	16 (26.7)	
Scottsdale/Phoenix	33 (27.5)	14 (23.3)	13 (21.7)	
Primary position at highest level of football, n (%)				
Offensive lineman	22 (18.3)	22 (36.7)	–	0.09
Defensive lineman	14 (11.7)	5 (8.3)		
Offensive back or receiver	36 (30.0)	14 (23.3)		
Linebacker	21 (17.5)	7 (11.7)		
Defensive back	23 (19.2)	12 (20.0)		
Special teams	4 (3.3)	0 (0.0)		
Total years of football, mean (SD)	18.0 (3.3)	11.5 (2.5)	–	< 0.01
Total fall seasons of college football, mean (SD)	4.1 (0.5)	3.9 (0.6)	–	0.11
Total years of NFL participation, mean (SD)	7.4 (2.7)	–	–	–
Age of first exposure to football, mean (SD)	11.5 (2.8)	10.2 (2.6)	–	< 0.01
Neuroimaging data available, n (%)				
Magnetic resonance imaging	114 (95.0)	55 (91.7)	56 (93.3)	–
Tau PET – Flortaucipir	112 (93.3)	58 (96.7)	58 (96.7)	
Amyloid PET – Florbetapir	119 (99.2)	60 (100.0)	58 (96.7)	
Biofluids collected, n (%)				
Cerebrospinal fluid	102 (85.0)	40 (66.7)	46 (76.7)	–
Blood (plasma, serum, whole blood)	118 (98.3)	58 (96.7)	58 (96.7)	
Saliva	120 (100.0)	60 (100.0)	58 (96.7)	
MoCA total raw score, mean (SD)	24.3 (3.5)	25.4 (3.2)	26.5 (2.3)	< 0.01^d^
Functional Activities Questionnaire – Informant Total, mean (SD)	3.9 (5.5)	3.2 (4.9)	0.4 (2.4)	< 0.01^e^
ApoE genotype – n (%) ε4 carriers^f^	33 (28.7)	20 (33.9)	11 (19.6)	0.22

^a^Categorical variables compared with chi-square or Fisher’s exact tests. Continuous variables compared with T-test or ANOVA (for normally distributed data) or Mann-Whitney U or Kruskal-Wallis tests (for non-normally distributed data). Significant ANOVA post hoc pairwise group comparisons examined with Student-Newman-Keuls test

^b^PRO=UE > COL

^c^PRO=UE < COL

^d^PRO=COL<UE

^e^PRO=COL>UE

^f^ApoE genotyping unavailable for 10 participants (5 PRO, 1 COL, 4 UE)

### Neuroimaging

Neuroimaging protocols completed include structural T1- and T2-weighted MRI, diffusion MRI (dMRI), resting-state fMRI, MRS, as well as molecular imaging with two PET tracers, florbetapir and flortaucipir. Imaging calibration and quality control (QC) procedures were completed for all sites prior to participant enrollment and throughout data acquisition. PET phantoms from Invicro were checked and each site was certified by the Invicro team using their standard protocols. Additional MRI and MRS harmonization and QC procedures were employed by the BWH Psychiatry Neuroimaging Laboratory (PNL) and Center for Clinical Spectroscopy (CCS) (see Supplementary Material, Additional File 1). The four MRI sequences were acquired in approximately 40 min. Total MRS scan time was 15 min, including shimming. Participants also completed florbetapir and flortaucipir PET scans and 214 participants completed both PET scans. Florbetapir scans were typically conducted first (n = 157 [73%] of the 214 participants who had both PET scans), with at least 12 h between the two scans. PET protocol length is described above.

### Fluid biomarkers

At the completion of all baseline evaluations, plasma, and CSF samples were shipped on dry ice overnight from VA Puget Sound to the University of Gothenburg, Sweden, where primary biomarker assays were conducted in batch. Primary fluid biomarkers include plasma and CSF measures of p-tau_181_, p-tau_217_, p-tau_231_, total tau, abeta_40_, abeta_42_, abeta_38_, glial fibrillary acidic protein (GFAP), NfL, soluble triggering receptor expressed on myeloid cells 2 (sTREM2), and soluble platelet-derived growth factor receptor beta (sPDGFRbeta). Supplemental assays will be conducted at VA Puget Sound and University of Washington and will include the following: CSF and plasma CNS-derived extracellular vesicle (EV) total tau and p-tau epitopes 181, 231, and 396; vascular endothelial growth factor-A (VEGF-A); basic fibroblast growth factor (bFGF); interleukins (ILs) 1alpha and beta, 7, and 17a; tumor necrosis factor (TNF)-a; monocyte chemotactic protein (MCP)-1; C-reactive protein (CRP); alpha-synuclein; CSF serum albumin ratio; and CSF catecholamines and indolamines and their precursors and metabolites. Additional CSF, plasma, and saliva biomarkers will be examined as new discoveries direct.

### Multidisciplinary Diagnostic Consensus Conferences (MDCC)

MDCCs were completed for all participants following their baseline evaluations using the provisional 2014 TES criteria [54]. MDCCs are being repeated for all baseline evaluations to derive new TES diagnoses using the recently published 2021 NINDS Consensus Diagnostic Criteria for TES [56], but without re-diagnosing other conditions. Only these new TES diagnoses will be used in baseline data analyses. MDCCs will be held following remote follow-up evaluations to determine any change in TES diagnosis (using the 2021 criteria) or in other neurodegenerative and/or psychiatric diagnoses.

### First NINDS Consensus Workshop to define the diagnostic criteria for TES

Refinement and validation of the 2014 research diagnostic criteria for TES [54] is an aim of the DIAGNOSE CTE Research Project. Since the time of the original 2014 publication, the TES criteria have been used in several ongoing research studies, including the UNITE study [55] and for the initial baseline evaluation MDCCs in this DIAGNOSE CTE Research Project. In April 2019, the *First NINDS Consensus Workshop to Define the Diagnostic Criteria for TES* was held in Phoenix, Arizona. The goal of the workshop was to evaluate and update TES criteria based on the following: (1) inter-rater reliability data from research studies (including baseline MDCC data from this project); (2) predictive validity data, both published [55] and unpublished, examining the relationship between the specific criteria and neuropathologically diagnosed CTE; (3) systematic review of CTE literature to date; and (4) expert opinion. A Modified Delphi approach was followed and included a first round of anonymous voting that took place during the Workshop, followed by three additional online voting rounds on revised criteria. Voting panelists included 20 clinician-researchers across a variety of disciplines (e.g., Neurology, Neuropsychology, Psychiatry, Physical Medicine and Rehabilitation, Neurosurgery), areas of expertise (e.g., neurodegenerative disease, TBI), and academic institutions (i.e., ten). The Delphi process was completed in January 2020 and a report on the new NINDS Consensus Diagnostic Criteria for TES was published in 2021 [56]. These new TES diagnostic criteria are intended for research purposes and not for clinical diagnosis. It is expected that the criteria will be further updated and revised through future NINDS Consensus Workshops as research in this field and on the criteria evolve and biomarker data become available.

## Discussion

This report provides a description of the methodology for the DIAGNOSE CTE Research Project, a multicenter, observational, cohort study designed to develop, refine, and validate in vivo biomarkers for CTE; characterize the clinical course and presentation of this tauopathy; identify potential risk factors; and refine and validate research diagnostic criteria for the clinical presentation associated with CTE (i.e., TES). This report also provides a description of the demographics of the sample, comprised of 120 former NFL players, 60 former college football players, and 60 unexposed same-age asymptomatic men.

Methodological decisions were made in designing the DIAGNOSE CTE Research Project based on the overarching goal of establishing clinical diagnostic criteria for CTE with highly accurate in vivo biomarkers. As such, we decided to focus on a sample of former college and professional American football players to ensure a sample at high risk for CTE [3] and to maximize power for hypothesis testing. Although the inclusion of a more heterogeneous sample of contact and collision sport athletes (e.g., boxers, soccer players, ice hockey players, including women), as well as participants with other sources of repetitive head impact exposure, such as military combat veterans with blast-injuries, survivors of intimate partner violence, and younger participants, may increase generalizability, it would be difficult to estimate “exposure” levels or achieve adequate statistical power or assure that the sample was at high risk for CTE. Thus, at the time of initial development of in vivo CTE diagnostics, homogeneity of the source of repetitive head impacts was prioritized. An area of active investigation by our team includes other, non-football contact sport athletes (e.g., soccer, ice hockey, rugby), particularly female former contact sport athletes. Some investigators of DIAGNOSE CTE are leading a new NIH-funded initiative (PI: Stern), known as the Head Impact and Trauma Surveillance Study (HITSS), that will leverage the online Brain Health Registry platform and recruit, enroll, and longitudinally follow female and male former soccer players and male former American football players (across all levels of play). This initiative will increase our understanding of the long-term effects of repetitive head impacts across sports and in females, and lead to future investigations that are similar to DIAGNOSE CTE, allowing for rich clinical characterizations of female former contact sport athletes.

The UE group was carefully selected. If the primary goal was to study disease risk, then certain variables should have been well-controlled, e.g., cardiovascular/cerebrovascular risks, performance enhancing drug use, substance use, history of team sport involvement. However, our primary goals were to examine possible biomarkers to detect CTE and the refinement of diagnostic criteria for the clinical manifestations of CTE. Therefore, our comparison group included individuals who were similar to the former American football players in terms of age, sex, and BMI, but did not have repetitive head impact exposure and were asymptomatic. This type of design is appropriate for biomarker development and validation. Importantly, while the UE group will allow us to answer questions regarding biomarker development and validation, their inclusion in other types of analyses requires careful consideration. For instance, it would be inappropriate to characterize the effect of repetitive head impact exposure on clinical measures in both the former player groups and UE group, given that the UE group was required to be asymptomatic (for neurological and psychiatric conditions) at the time of recruitment and the former players were not. Even among questions pertaining to biomarkers, it will be important to conduct sensitivity analyses to determine if any potential group differences are related to exposure to repetitive head impacts or to other factors. Lastly, other types of “control” groups were considered, but not incorporated into the design of the study. There have been efforts to recruit former professional baseball players or body builders as controls for similar studies, because they have similar lifestyles and body habitus as former American football players. Yet, there have been very few who had never participated in organized contact sports or who, in the case of baseball players, had not reported multiple concussions. Planned ancillary studies will also recruit participants with AD dementia and AD-related dementias as disease comparison groups to the former profession American football players.

All participants were required to have a study partner who knows them well to provide assessments of their perspective of the participant’s cognitive, behavioral, and functional status. In some cases, these reports may be inaccurate due to a variety of factors, including misattributions of symptoms to age-related changes or stress; exaggeration of deficits for potential secondary gain (including financial compensation from disability or legal cases); and denial/unawareness of deficits (including anosognosia) due to neurodegenerative disease and other neurologic conditions. This requirement may introduce some degree of selection bias due to the potential for some participants with underlying CTE to have neuropsychiatric features (e.g., rage, aggression, impulsivity) that result in the loss of close relationships and overall social isolation. Therefore, it is possible that potential participants with more severe neuropsychiatric features were excluded due to those features limiting the availability of a study partner.

The primary method of validating biomarkers for the detection of CTE pathology or to truly examine risk factors for the development of CTE pathology is to compare data collected during life with postmortem neuropathology and diagnosis. The large majority of former college and professional football players in the DIAGNOSE CTE cohort have agreed to brain donation. At the time of the current paper, five former players had already died, and their brain tissue will be examined for these clinicopathological correlation and validation studies.

Our goal was to enroll a similar proportion of Black participants across the three exposure groups (i.e., PRO, COL, UE), with the target of approximately 40% overall. Although the PRO and UE groups have a similar proportion of Black participants, with 42.5% and 40.0%, respectively, the COL group has a significantly smaller proportion of black participants (16.7%). Interactive effects between levels of exposure to repetitive head impacts and Black racial identity on potential neuroimaging and fluid biomarkers of CTE have been reported [161]. Moreover, there are potential differences between Black and White participants in the expression of psychiatric symptoms and performance on cognitive tests [175, 176], as well as important racial disparities in life-course social determinants of health, cognitive aging, and neurodegenerative disease [177–179]. For these reasons, interpretation of data analyses including the COL group will be done with these racial identity differences in mind [180].

## Conclusions

The DIAGNOSE CTE Research Project will lead to a rich dataset that will be used to further our understanding of CTE in terms of its clinical presentation, in vivo biomarkers, clinical research diagnostic criteria, and risk and resiliency factors for the development of CTE. In addition to repetitive head impact exposure and genetic factors, project data will inform on the role of demographic, lifestyle, medical, and psychiatric risk and resilience variables, as well as on social determinants of health and racial disparities. Importantly, the data will provide the infrastructure and resources for opportunities to conduct ancillary or add-on studies that target questions not being directly examined by the DIAGNOSE CTE Research Project. Ultimately, it is anticipated that findings from the DIAGNOSE CTE Research Project and associated ancillary studies will facilitate the ability to detect and diagnose CTE during life and thereby accelerate research on risk factors, mechanisms, epidemiology, and, most importantly, treatment and prevention of CTE.

## Supplementary Information

**Additional file 1.**

## Data Availability

The datasets generated and analyzed during the current study will be available in the Federal Interagency Traumatic Brain Injury Research (FITBIR) repository, https://fitbir.nih.gov. Datasets will also be available through a data sharing portal for the DIAGNOSE CTE Research Project, http://diagnosecte.com. It is also anticipated that study datasets will be available in the Global Alzheimer’s Association Interactive Network (GAAIN) repository, http://www.gaain.org.

## Abbreviations

3R: Three microtubule-binding domain repeat tau isoform
4R: Four microtubule-binding domain repeat tau isoform
Aβ: Beta-amyloid
AD: Alzheimer’s disease
AxD: Axial diffusivity
BAI: Banner Alzheimer’s Institute
BMI: Body mass index
BU: Boston University
BUMC: Boston University Medical Campus
BUSM: Boston University School of Medicine
BWH: Brigham and Women’s Hospital
CC: Cleveland Clinic
CDE: Common Data Elements
CSF: Cerebrospinal fluid
COL: Former college player group
CTE: Chronic traumatic encephalopathy
DEI: Diversity, equity, and inclusion
DIAGNOSE CTE: Diagnostics, Imaging, and Genetics Network for the Objective Study and Evaluation of Chronic Traumatic Encephalopathy
dMRI: Diffusion MRI
DTI: Diffusion tensor imaging
FA: Fractional anisotropy
FITBIR: Federal Interagency Traumatic Brain Injury Research
FLAIR: Fluid attenuated inversion recovery
fMRI: Functional magnetic resonance imaging
GWAS: Genome-wide sequencing studies
IRB: Institutional Review Board
LP: Lumbar puncture
MD: Mean diffusivity
MDCC: Multidisciplinary diagnostic consensus conference
MDS: Movement Disorder Society
MRI: Magnetic resonance imaging
MRS: Magnetic resonance spectroscopy
NFL: National Football League
NFT: Neurofibrillary tangle
NINDS: National Institute for Neurological Disorders and Stroke
NYU: New York University
PET: Positron emission tomography
PI: Principal investigator
PNL: Psychiatry Neuroimaging Laboratory
PRO: Former profession (NFL) player group
p-tau: Hyperphosphorylated tau
QC: Quality control
RD: Radial diffusivity
SNP: Single-nucleotide polymorphism
TBI: Traumatic brain injury
TES: Traumatic encephalopathy syndrome
t-tau: Total tau
UDS: Uniform Data Set
UE: Unexposed group
UNITE: Understanding Neurologic Injury in Traumatic Encephalopathy
