# Supplementary material for: Developing methods to detect and diagnose chronic traumatic encephalopathy during life: rationale, design, and methodology for the DIAGNOSE CTE Research Project
Source: Alzheimers Res Ther. 2021 Aug 12;13:136. doi: 10.1186/s13195-021-00872-x (PMC8357968; doi:10.1186/s13195-021-00872-x)
Supplement: Supplementary file 1 — Additional file 1. [file 13195_2021_872_MOESM1_ESM.pdf]

Developing Methods to Detect and Diagnose Chronic Traumatic Encephalopathy During Life: Rationale, Design, and Methodology for the DIAGNOSE CTE Research Project

Michael L. Alosco, Ph.D., Megan L. Mariani, B.S., B.A., Charles H. Adler, M.D., Ph.D., Laura J. Balcer, M.D., M.S.C.E., Charles Bernick, M.D., Rhoda Au, Ph.D., Debra Babcock, M.D., Ph.D., Sarah J. Banks, Ph.D., William B. Barr, Ph.D., Sylvain Bouix, Ph.D., Robert C. Cantu, M.D., Michael J. Coleman, M.A., David W. Dodick, M.D., Lindsay A. Farrer, Ph.D., Yonas E. Geda, M.D., Douglas I. Katz, M.D., Inga K. Koerte, M.D.,Ph.D., Neil W. Kowall, M.D., Alexander P. Lin, Ph.D., Daniel S. Marcus, Ph.D., Kenneth L. Marek, M.D., Michael D. McClean, Sc.D., Ann C. McKee, M.D., Jesse Mez, M.D., M.S., Joseph N. Palmisano, M.A., M.P.H., Elaine R. Peskind, M.D., Yorghos Tripodis, Ph.D., Robert W. Turner II, Ph.D., Jennifer V. Wethe, Ph.D., Jeffrey L. Cummings, M.D., Sc.D., Eric M. Reiman, M.D., Martha E. Shenton, Ph.D., Robert A. Stern, Ph.D., for the DIAGNOSE CTE Research Project Investigators

Funded by a grant from the National Institute of Neurological Disorders and Stroke of the National Institutes of Health (U01NS093334)

Supplementary Materials

Additional File 1

Contents

Supplementary Table 1. Collaborating Institutions 2
Supplementary Table 2. External Advisory Board 3
Recruitment and Retention 4
Neuroimaging Harmonization and Quality Control Procedures 7
MRI Processing and Analysis 9
MRS Processing and Analysis 10
Baseline Saliva and Blood Collection Procedures and Sample Processing 11
Lumbar Puncture (LP) Procedure and Cerebrospinal Fluid (CSF) Processing 12
Follow-Up Blood Collection Procedures and Sample Processing 13
Management and Sharing of Data and Biospecimens 14
References for Supplementary Materials 16

Supplementary Table 1. Collaborating Institutions

The DIAGNOSE CTE Research Project Collaborating Institutions
Banner Alzheimer's Institute, Phoenix, AZ
Boston University Schools of Medicine and Public Health, Boston, MA
Brigham and Women's Hospital, Boston, MA
Cleveland Clinic Lou Ruvo Center for Brain Health, Las Vegas, NV
Invicro (formerly Molecular NeuroImaging), New Haven, CT
Mayo Clinic, Scottsdale, AZ
National Institute of Neurological Disorders and Stroke (NINDS),* Bethesda, MD
New York University Langone Health, New York, NY
Seattle Institute for Biomedical and Clinical Research (SIBCR), VA Puget Sound Health Care System, Seattle, WA
University of Nevada School of Integrated Health Sciences, Las Vegas, NV
University of Washington, Seattle, WA
Washington University, St. Louis, MO

*This project is funded by NINDS through a U01 Research Project Cooperative Agreement

Supplementary Table 2. External Advisory Board

**Board Member**	**Affiliation**
David S. Knopman, M.D., Chair	Mayo Clinic (Rochester, MN)
Colonel (Ret.) Dallas C. Hack, MD, MPH	Independent Consultant (Previously Director, Combat Casualty Care Research Program, U.S. Army)
Brian Hainline, M.D.	National Collegiate Athletic Association
Mike Haynes	Member of the Pro Football Hall of Fame
Thomas W. McAllister, M.D.	Indiana University School of Medicine
Arthur W. Toga, Ph.D.	University of Southern California
Michael W. Weiner, M.D.	University of California, San Francisco

Recruitment and Retention

Enrollment began in September 2016. Recruitment and telephone screening activities were centralized at the BU Coordinating Center. A full-time Recruitment and Retention Coordinator with a full-time research assistant oversaw an extensive national recruitment campaign, including: a study-specific website, with targeted 90-second videos of former Super Bowl Champion, Ben Utecht; social media (Twitter, Facebook) account posts; newspaper ads geared toward potential unexposed (UE) participants in urban and suburban newspapers in Boston, Scottsdale/Phoenix, and New York City; webinar and newsletter posts through the Alzheimer's Prevention Registry; paper flyer distribution through in-person community event tabling and postings at fitness centers, barbershops, etc.; electronic distribution of flyers through blog posts by former NFL players, via the NFL Wives Facebook Group, and through email distributions to NFL alumni groups and college alumni associations; postcard mailings through the US Postal Service's Every Door Direct Mail program; posters in commuter rail system train cars; large digital screen/billboard displays inside Boston's South Station and outside New York's Penn Station; public radio underwriting spots in select markets around the country; several searches through ResearchMatch (a nationwide online database of research volunteers); and through word of mouth, including from study participants. We had an initial goal of enrolling a similar proportion of Black participants across the three exposure groups, with the target of approximately 40%, based on the proportion of Black former NFL players who played during the years our sample would have played (1967-1996). Specific recruitment activities targeting Black former college players and potential UE participants were implemented, including: requests sent to Historically Black Colleges and Universities; radio ads in the Atlanta

metro area; “Railcards” in Prince George’s County and other Washington DC Metro stations; and targeted outreach by community leaders involved with the project; and through word of mouth, including from study participants. However, based on the telephone screening scripts, it was not possible to know which of the many approaches were most successful in leading individuals to contacting the Coordinating Center. As shown in the demographics table (Table 5 of the manuscript) and summarized in the Discussion, our recruitment efforts resulted in us reaching our target of approximately 40% Black/African American enrollment in the former NFL player group and the unexposed control group, but despite extensive and varied outreach approaches, we were unable to achieve that target in the former college player group.

To maximize sample retention over the four-year follow-up period, participants are telephoned annually by project staff and sent birthday and holiday cards. A study-wide newsletter is published quarterly and distributed to all participants electronically. For participants with dementia, an additional annual call is made to either the participant or (with permission) to a study partner/informant to assess the participant’s status and improve retention. At the time of the COVID-19 pandemic and the change to remote follow-up evaluations, additional retention activities were initiated. Each participant was contacted via telephone from the study team to provide them with the most up-to-date project information and to answer questions. “Care packages” containing hand sanitizer, a stress ball (with project logo), study-relevant word-game worksheets, and appointment reminders, are sent out prior to participants’ scheduled follow-up evaluations. A brochure highlighting information regarding the remote follow-up evaluation and associated changes to the study is distributed. Online participant surveys are conducted, allowing for the collection of valuable participant data while simultaneously monitoring response rates. An infographic detailing the in-home blood draw process is distributed. Webinars (live with

available recordings) are provided specifically for participants and study partners periodically throughout the follow-up period, with topics addressing issues relevant to them (e.g., “Can we diagnose CTE during life yet” “Sleep and brain health”). There is also ongoing collaboration between the Retention Coordinator and the Diversity, Equity, and Inclusion Team to assure appropriate and engaging messaging to all participants.

Neuroimaging Harmonization and Quality Control Procedures

MRI and MRS phantoms were constructed at the Brigham and Women's Hospital (BWH) Psychiatry Neuroimaging Laboratory (PNL) and distributed to the four sites. PNL investigators travelled to each site to install protocols and test them with the phantom for quantitative assessment of each scanner. Moreover, the same four members of the imaging team were scanned with the complete protocol at each site as "traveling heads". These data were used for QC and data harmonization along with the phantom data. Two traveling heads were then scanned annually at each site and phantom scans were acquired each quarter. MRI QC was conducted on the phantom and traveling heads to confirm consistency of acquisition parameters, signal-to-noise and contrast-to-noise ratio across modalities, as well as geometric features of images across scanners. Images were also inspected for any major artifact. MRS QC was conducted on the phantom and traveling head data to check for any major artifacts and that metabolite levels remained consistent from site to site and from year to year. Traveling head data were used for harmonization of data across sites and to ensure that there was no evidence of spectral drift from year to year.

MR imaging data acquired at the four Performance Evaluation Sites were initially imported via a secure web-based upload tool to the Central Neuroimaging Data Archive, an XNAT-based data management platform operated by the Neuroinformatics Research Group at Washington University. PET data from the four sites were uploaded directly to Invicro where QC procedures were conducted for each scan at the time of upload. Sites were notified immediately if there were issues with QC or with other aspects of PET acquisition. For the baseline evaluations, no-recalibration actions were needed. QC checks for MRS data received

from each site included: 1) consistent acquisition parameters; 2) appropriate voxel location and volume; 3) SNR measurement of > 10 for SVS voxels (8 cm^3) and > 2 for CSI voxels (1.5 cm^3); and 4) linewidth measurement of less than 15 Hz in SVS data and < 20 Hz in CSI data. In the post-processing stage of the MRS data, Cramer-Rao lower bounds were checked for each metabolite ranging from 1% to no greater than 20%.

MRI Processing and Analysis

A combination of automated and semi-automated processing pipelines were used to derive the basic macrostructural indices from the MRI data. For structural MRI, brain masking was performed using a tool developed at the BWH PNL (1), then gross and regional gray and white matter volumes, and cortical thickness were extracted using FreeSurfer 7.1. The fluid-attenuated inversion recovery (FLAIR) sequences were analyzed to estimate total and regional volume of white matter hyperintensities using the automated lesion prediction algorithm (LPA) pipeline from the Lesion Segmentation Toolbox (LST) for SPM in MATLAB (MathWorks, Natick, MA) (2). For dMRI analyses, images were brain masked using a deep learning-based brain masking tool that robustly extracts the brain from dMRI data in a few seconds on a graphics processing unit (GPU)(3). The data were then corrected for Gibbs ringing artifacts, as well as geometric distortions due to participant motion, EPI acquisition, and eddy currents using a custom pipeline (4). The resulting corrected dMRI data were harmonized to remove site effects using a retrospective harmonization algorithm (5). Diffusion measures were estimated at each voxel based on the diffusion tensor model resulting in four diffusion measures: Axial Diffusivity (AxD), Fractional Anisotropy (FA), Radial Diffusivity (RD), and Mean Diffusivity (MD). For resting state fMRI data, metrics included traditional seed-to-target within- and between network interactions.

Additional analyses will include the evaluation of cavum septum pellucidum presence and length from T1w images. For dMRI, Tract Based Spatial Statistics will be performed for AxD, RD, FA, and MD. Additionally, the integrity of the white matter at the depths of the cortical sulci will be examined, and whole brain tractography will be performed with tracts of interest automatically segmented using tools developed at the BWH PNL (6, 7).

MRS Processing and Analysis

The 2D-chemical shift imaging (CSI) data and single voxel spectroscopy (SVS) data were pre-processed using the OpenMRSLab to include frequency, phase, and eddy current correction (8) and post-processed using LC-Model software (9). Given the predicted signal-to-noise ratio (SNR), we expected reliable (Cramer-Rao lower bounds < 15%) absolute quantification for N-acteyl aspartate (NAA), creatine (Cr), choline (Cho), glutamate (Glu), glutathione (GSH), and myo-inositol (mI). Within the OpenMRSLab platform the T1 MPRAGE images were segmented using FSL (10) and co-registered to the 2D-CSI and SVS voxels. Each of the spectroscopic voxels was assigned a percentage volume of gray and white matter or other brain tissue type. This allowed for the evaluation of metabolites' content spatially, CSF correction, absolute quantitation of the SVS MRS voxel, and for correlation of metabolic amounts with different brain tissue types for the 2D-CSI.

Baseline Saliva and Blood Collection Procedures and Sample Processing

Participants were instructed to fast at least 12 hours prior to fluid biomarker collection, with no eating, drinking, or use of oral hygiene products at least 1 hour prior to the saliva collection (first fluid biomarker procedure). Participants were provided a 15mL saliva collection tube on ice and were instructed to passively drool. The procedure was stopped after 20 minutes or until 5mL was obtained, whichever came first. The saliva sample was processed by adding the appropriate amount of 10X PIC and centrifuging the sample within 20 minutes of collection at 2000g for 15 minutes at 4ºC and transferred into 25 aliquot tubes (0.5mL tubes).

Following saliva collection, an IV was placed for blood collection and the participant rested lying down for at least 30 minutes prior to blood collection. The first 3cc of saline-diluted blood were discarded and five 10cc syringes of blood were then collected and transferred into collection tubes (3mL Cat, 3x7.5mL serum, 4x10mL regular plasma, 5x10mL platelet-free plasma, 3x10mL whole blood). A portion of whole blood (2x10mL) was kept at room temperature and shipped to BUSM the day of collection (for DNA extraction, APOE genotyping, and storage for later GWAS and other genetic and genomic analyses). All other blood samples were centrifuged at 1180g for 15 minutes at 4ºC, aliquoted, and stored at -70ºC within 90 to 120 minutes of collection.

Lumbar Puncture (LP) Procedure and Cerebrospinal Fluid (CSF) Processing

The LP was performed by a qualified physician, physician assistant, or nurse practitioner, with the participant placed in a lateral decubitus or upright sitting position, using a Sprotte atraumatic needle. A 20g spinal introducer needle was inserted through the interspace of L3-4 or L4-5, and a 24g Sprotte (90mm or 123mm) was inserted through the introducer. The initial 1-2mL of CSF was discarded if not visibly clear, and sterile 6cc syringes were used until 20mL of CSF was collected. To reduce the risk of post-LP headache, participant's rested on their back or side for one hour after the LP procedure was completed. For initial processing of CSF samples, the first 2mL of CSF were aliquoted into a 15mL tube for clinical labs and kept at room temperature. The remaining CSF was aliquoted into 0.5mL tubes (except 1.0mL tubes for catecholamines) and placed on dry ice.

Follow-Up Blood Collection Procedures and Sample Processing

The BU Coordinating Center contracted a remote phlebotomy vendor, ExamOne, to collect blood from all consenting participants at their homes at the time of their four year follow-up evaluations. ExamOne is a Quest Diagnostics company that provides nationwide mobile phlebotomy collections in the homes of their individual clients. To assure consistency and QC of the blood collection procedures and sample processing, a smaller group of experienced phlebotomists have been selected by ExamOne for this study who undergo study-specific training. Pre-fabricated blood collection and sample preparation supplies, along with a manual and infographic detailing all procedures, are shipped to the participant's home for use by the phlebotomist. Fasting blood draw via venipuncture is conducted in the morning, with 3x7.5mL serum, 4x10mL plasma, and 1x6mL whole blood collection tubes. The whole blood tube is kept at room temperature and all other tubes are placed in wet ice until centrifugation. Serum and plasma samples are centrifuged at 1600g for 12 minutes. All samples are aliquoted and put on dry ice within 90 minutes of centrifugation, and shipped to VA Puget Sound, where they are banked for biomarker assays and distribution to qualified investigators.

Management and Sharing of Data and Biospecimens

Baseline evaluations resulted in over 10 thousand data points and hundreds of biospecimens per participant, requiring a complex, highly integrated system to manage the capture, delivery, and sharing of these data. The Biostatistics and Epidemiology Data Analytics Center (BEDAC) at the BU School of Public Health provides data management, database and web development, and data analysis for the project (the latter in collaboration with the project's lead biostatistician and Data Team Leader (YT). Throughout the project, there has been close interaction between the Data Team, the BU Coordinating Center, and study investigators and staff at all sites, resulting in procedural consistency, efficiency of the study-wide systems, and ultimately high quality data. Data collection strategies include web-based data capture for assessments using REDCap, as well as customized forms for complex data. A web-based tracking system was developed to track participants, data, and specimens. Data collection and management systems were created for all aspects of the project. When possible, data are collected on tablet or laptop computers. Electronic forms include required fields, skip patterns, and ranges, resulting in more complete and "cleaner" data at capture. All data, regardless of capture method, are converted to SAS datasets and reviewed for logic, skip patterns, response ranges, and internal inconsistencies. The project databases are located on a secure server within the BU Medical Campus domain. All web-forms are protected using secure socket layering encryption technology and protected by electronic 'firewalls' that restrict access to designated users. Data are transmitted to and from BU via a secure and encrypted system. Additionally, common data elements and study-specific data elements are uploaded to FITBIR on a regular basis to allow for data sharing in the latter part of the project.

Once baseline data collection was completed, the Data Team developed a web-based data

sharing platform, initially for use by project investigators, with the plan for subsequent availability to all qualified researchers (i.e., in the latter part of the project). This includes a REDCap-based system to facilitate the data request and approval process. For baseline data alone, there are over 100 domain level baseline analytic datasets, each with a detailed data dictionary. Based on the specific needs of an investigator, a customized dataset is created using an automated system. Raw imaging data and fluid biosamples will also be made available to qualified investigators. External investigators requesting fluid biosamples will be required to provide a detailed proposal that will undergo a more extensive review process due to the limited and finite quantity of samples.

References for Supplementary Materials

1. Del Re EC, Gao Y, Eckbo R, Petryshen TL, Blokland GA, Seidman LJ, et al. A New MRI Masking Technique Based on Multi-Atlas Brain Segmentation in Controls and Schizophrenia: A Rapid and Viable Alternative to Manual Masking. Journal of neuroimaging : official journal of the American Society of Neuroimaging. 2016;26(1):28-36.

2. Schmidt P, Gaser C, Arsic M, Buck D, Förschler A, Berthele A, et al. An automated tool for detection of FLAIR-hyperintense white-matter lesions in Multiple Sclerosis. NeuroImage. 2012;59(4):3774-83.

3. Palanivelu S, Cetin Karayumak S, T B, S B, Y R. CNN based diffusion MRI brain segmentation tool 2020 [Available from: https://github.com/pnlbwh/CNN-Diffusion-MRIBrain-Segmentation.

4. Billah T, Bouix S. A Luigi workflow joining individual modules of an MRI processing pipeline 2020 [Available from: https://github.com/pnlbwh/luigi-pnlpipe.

5. Cetin Karayumak S, Bouix S, Ning L, James A, Crow T, Shenton M, et al. Retrospective harmonization of multi-site diffusion MRI data acquired with different acquisition parameters. NeuroImage. 2019;184:180-200.

6. Zhang F, Wu Y, Norton I, Rigolo L, Rathi Y, Makris N, et al. An anatomically curated fiber clustering white matter atlas for consistent white matter tract parcellation across the lifespan. NeuroImage. 2018;179:429-47.

7. Malcolm JG, Shenton ME, Rathi Y. Filtered multitensor tractography. IEEE transactions on medical imaging. 2010;29(9):1664-75.

8. Alosco ML, Tripodis Y, Rowland B, Chua AS, Liao H, Martin B, et al. A magnetic resonance spectroscopy investigation in symptomatic former NFL players. Brain imaging and behavior. 2020;14(5):1419-29.

9. Provencher SW. Automatic quantitation of localized in vivo 1H spectra with LCModel. NMR Biomed. 2001;14(4):260-4.

10. Jenkinson M, Beckmann CF, Behrens TE, Woolrich MW, Smith SM. FSL. NeuroImage. 2012;62(2):782-90.
